# Level of anxiety symptoms and its associated factors among nurses working in emergency and intensive care unit at public hospitals in Addis Ababa, Ethiopia

**DOI:** 10.1186/s12912-021-00701-4

**Published:** 2021-09-26

**Authors:** Zelalem Belayneh, Abriham Zegeye, Eshetu Tadesse, Biksegn Asrat, Getnet Ayano, Birhanie Mekuriaw

**Affiliations:** 1 College of Health and Medical Science, Department of Psychiatry, Dilla University, Dilla, Ethiopia; 2 Department of Biomedical Sciences, School of Medicine, Debre Markos University, Debre Markos, Ethiopia; 3 Research and Training Department, Amanuel Mental Specialized Hospital, Addis Ababa, Ethiopia; 4 Department of Psychiatry, School of Medicine, College of Medicine and Health Science, University of Gondar, Gondar, Ethiopia

**Keywords:** Anxiety, Distress, Work stress, Emergency Nurses, Job satisfaction

## Abstract

**Background:**

Anxiety is a common phenomenon in some professions including medical emergency settings. Nurses deal with grief and other psychological disturbances when they lost clients due to death at clinical settings. Thus, the level of anxiety among nurses working at emergency and intensive care unit is expected to higher as a result of life threatening cases and frequent loss of clients at emergency settings. However, the burden of anxiety and its associated factors among nurses working in emergency clinical settings are not well addressed in Ethiopia.

**Methods:**

An institutional based cross-sectional study design was conducted among 415 randomly selected nurses working at emergency and Intensive Care Unit at public hospitals in Addis Ababa. Data were collected using interviewer administered questioner. The Hamilton Anxiety Rating Scale was used to measure level of anxiety symptoms. The collected data were entered to a computer using Epi-Data Version 3.1 and exported to SPSS Version 20.0 for analysis. Binary logistic regression was used to identify factors associated with anxiety. Variables with *P*- Values of < 0.05 were considered as having statistically significant association with higher level of anxiety symptoms with 95 % confidence intervals.

**Results:**

The result of this study shows that 19.8 % nurses working at emergency and intensive care unit had a higher level of anxiety symptoms [95 % CI (16.1 %- 23.6 %)]. Marital status{0.28:95 %CI(0.16–0.50)}, cigarette smoking{2.48:95 %CI(1.18–5.18)}, work overload {0.35:95 %CI(0.16,0.76)} and night duty shift{0.41:95 %CI(0.19–0.87)} were factors significantly associated with higher level of anxiety symptoms among nurses working at emergency medical settings.

**Conclusions:**

Nurses working at emergency and intensive care unit showed higher level of anxiety symptoms than the general population and nurses working at other medical settings. Marital status, cigarette smoking, work overload and night duty shift had statistically significant association with higher anxiety symptoms among nurses working at emergency medical settings. This demonstrates a need for the implementation of counseling services regarding effective coping mechanisms and problem-solving strategies for nurses working at emergency medical settings.

**Supplementary Information:**

The online version contains supplementary material available at 10.1186/s12912-021-00701-4.

## Background

Anxiety is a psychological and physiological state characterized by cognitive, somatic, emotional, and behavioral components shown by unpleasant feelings, uneasiness, apprehension, fear, or worry, sleep disturbances and higher states of autonomic stimulation [1]. Anxiety can be explained as a normal reaction to stressful situations and may help people to deal with stressful or threatening situations. However, if anxiety could not be managed on time and properly, it may result in pathological conditions often accompanied by headache, sweating, muscle spasms, palpitations, fatigue or even exhaustion. Pathological anxiety is excessive and persistent emotional disturbance that affects the functionality, behavior, relationships and overall quality of life of people [2].

Anxiety symptoms are commonly observed phenomenon in some work places, particularly at emergency medical settings. The largest workforce in any health care institution is encompassed by nurses. Nurses are dedicated with the care of individuals, families, and communities to attain, maintain, or recover optimal health and quality of life of their clients [3, 4]. Nurses are much closer and spend more time with clients than other health care workers, even at night. This requires to pay a high level of attention to patient’s safety in such complex and life saving environments [5]. Therefore, nurses frequently deal with grief and become anxious when they lost their clients due to death at clinical settings. The level of anxiety symptoms and dissatisfaction is expected to higher among nurses working at emergency and ICU where life threatening cases are intervened and loss of clients is most frequently happened [6].

Evidences suggest that several work-related stresses, dissatisfaction, grief and anxiety at emergency and Intensive Care Unit (ICU) diminishes the quality care delivered [7, 8], treatment outcome [8, 9] patient and family satisfaction and the overall quality of health care services [10, 11]. This may also lead to occupational accidents such as medication errors, needle-stick injuries, and incorrect operation of medical equipment [12, 13] as a result of the sleep disturbances, wornness or fear, hopelessness and other behavioral symptoms which are considered as hallmark symptoms of anxiety disorders [10, 14].

Reduction of anxiety symptoms and emotional exhaustion among nurses working at emergency and ICU can be a wise remedy to reduce staff turnover rates and improve the quality of care and staff welfare of clients [15]. This can improve the quality of health care services and treatment outcome of people with critical health conditions [16]. However, there is scarcity of evidences regarding the level of anxiety and its associated factors among nurses working at emergence health care units in Ethiopia. Instated, the focuses of most studies is among health professionals in general and nurses working other than emergency settings. Thus, there is a great demand of evidence regarding the level of anxiety symptoms among emergency and ICU nurses, specifically. These populations are working at stressful environments where life threatening cases and death is very common. Therefore, this study was aimed to assess the level of anxiety symptoms and its associated factors of among nurses working at emergency and intensive care units at Addis Ababa public hospitals. The finding of this study will be helpful for health professionals, officials, governmental and non-governmental organization. Moreover, the finding of this study will be used as baseline information for further research works.

## Methods

### Study setting, design and period

This was a cross-sectional study conducted among nurses working in emergency and ICU settings of public hospitals at Addis Ababa (the capital city of Ethiopia) from April 01–30, 2017. In Addis Ababa, there are about 11 public hospitals where a total of 1,484 nurses are permanently employed. The ratio of all nurses to Addis Ababa population is approximately 1 to 942 according to the Ethiopian population growth estimation of 2011 EC.

### Population

Permanently employed nurses (having at least 6-months of relevant work experience) working in emergency and intensive care units at public hospitals of Addis Ababa are participated in this study Nurses working at other than emergency health care settings, volunteer service providers, nurses with known mental problems and having work experience of less than 6 months were excluded from the study.

### Sample size determination and sampling method

A single population proportion formula was used for sample size calculation with assumptions of a 95 % confidence interval (CI), proportion of anxiety among nurses 50 % and precision of 5 % and 10 % of non-response rate. Finally, the calculated total sample size became 423.

A systematic random sampling technique was employed to select study participants using record identification numbers. Initially, the total number of nurses working at emergency and ICU was found from each hospital. Then, the calculated sample size was proportionally allocated to each hospital based on the population size. Finally, individual participants were identified through intervals (K). The sampling interval (K) was calculated by dividing the total number of nurses working in emergency and ICU at each hospital to the sample size allocated to be drawn from that hospital. The first participant was selected by lottery method between one and K. K value was subsequently added to select the next respondent until the allocated sample has been addressed.

### Data collection instruments and procedures

Data were collected using interviewer administered questionnaire having different sub-sections including socio-demographic factors (age, sex, marital status, educational level, years of work experience), habit of substance use, work area related characteristics (type of critical care settings, work load, type of shift, satisfaction level and intention to change work within the next 12 months) and the Hamilton Anxiety Rating Scale (HARS) (Supplementary file 1).

The questionnaire was first prepared in English and translated to Amharic (the local language of the study setting), and then back translation to English was done to maintain its consistency. The questionnaire was pretested at Debre Markos Referral Hospital among 22 nurses working at emergency and ICU. Based on the findings of the pretest, minor modification was done on the questionnaires and some statements were rephrased to make easily understandable by study participants.

The level of anxiety symptoms was measured using the Hamilton Anxiety Rating Scale. The tool is one of the first rating scales developed to measure the severity of anxiety symptoms, and it is still widely used both in clinical and research settings. The HARS consists of 14 item likert scale questions. Each question is defined by a series of anxiety symptoms used to measure both psychic anxiety (mental agitation and psychological distress) and somatic anxiety (physical complaints related to anxiety) [17]. Questions have five different response options (0 = not present to four = very severe). The total score of each respondent is added together independently and individuals with a higher sum score were considered as having more severe anxiety symptoms and vice versa. In this study, a total HARS sum score of 8 and above is considered as a cutoff point to define higher level of anxiety symptoms. This cut point has been used in different studies and showed acceptable reliability. Although the tool is not validated in Ethiopian context, we have properly translated and questions have been rephrased with the cultural context of the study population.

Six BSc psychiatric nurses (data collectors) and two MSc level mental health professionals (supervisors) were participated in the data collection after attending two days of training. First, each eligible individual was requested to provide consent of participation after a brief explanation regarding the purpose and scopes of the study. After securing consent of participation, psychiatric nurses read each question one by one and respondents were requested to respond for each question among the given five alternatives ranging from 0 (Not present) to 4 (Very severe).

Job satisfaction was measured with a simple question of “In general, are you being satisfied with your job?”with three possible answers (satisfied, neutral and dissatisfied). The collected data was reviewed for its completeness and consistency by data collection supervisors and the principal investigator on daily base. Questions with incomplete data were excluded from the analysis.

### Data processing and analysis procedures

The collected data was first checked for its completeness and consistency. The data was then entered to Epi-data version 3.1 and exported to SPSS version 20.0 for analysis.

Frequency, percentages and tables were applied to report sociodemographic variables and the level of anxiety symptoms. Binary logistic regression was computed to identify factors associated higher level of anxiety symptoms. In the bi-variable analysis, variables with *P* < 0.25 were considered as eligible candidates for further multivariable analysis to avoid cofounders. Then, variables fulfilling the criteria were entered together to the multivariable model and *P*- values were used to determine the level of significance. In the final model, variables with *P*-values of < 0.05 were considered as having statistically significant association with higher level of anxiety symptoms. Odds ratio with 95 % confidence intervals was used to estimate the strength of the association.

## Results

### Socio-demographic characteristics of respondents

From a total of 423 nurses invited to participate in the study, 415 completed the interview with a response rate of 98.1 %. Majority (62.9 %) of respondents were females. The age of respondents ranges from 22 to 48 years old with the mean and standard deviation of 29.5 (SD = 6.4) years (Table 1).

**Table 1:** Sociodemographic characteristics of nurses working at emergency and ICU at Addis Ababa public hospitals, 2017 (*n* = 415)

Variables	Categories	Frequency/Percentage
**Sex**	Male	154(37.1)
	Female	261(62.9)
**Religion**	Orthodox	287(69.1)
	Muslim	42(10.1)
	Protestant	52(12.8)
	Catholic	34(8.0)
**Marital status**	Single	193(46.5)
	Married	216(52.1)
	Separated/Divorced	6(1.4)
**Employing Institution**	St. Powulos	55(13.3)
	St.Petrose	13(3.1)
	Tikur Anbesa	143(34.5)
	Zewditu	42(10.1)
	Yekatit 12	34(8.2)
	Minilik	20(4.8)
	ALERT	19(4.6)
	Gandi	18(4.3)
	Trunesh Beijing	23(5.5)
	Ras desta	27(6.5)
	Amanuel	21(5.1)
**Ethnicity**	Oromo	81(19.5)
	Amhara	207(49.9)
	Tigrae	64(15.4)
	Guragae	45(10.8)
	Others	18(4.3)
**Service area**	Emergency	172(41.4)
	ICU	243(58.6)
**Educational qualification**	Diploma nurse	84(20.2)
	BSc nurse	320(77.1)
	MSc nurse	11(2.7)

Abribbations: *BSc* Bachelor of Science, *ICU* Intensive Care Unit, *MSc* Master of Science

### Work related and clinical characteristics

About 20.7 % of nurses working at emergency and ICU mentioned that they have work overloads. Regarding the personal characteristics of respondents, 20.9 %, 23.4 % and 20.0 % of nurses had history of cigarette smoking, Kat chewing and alcohol use habit, respectively (Table 2).

**Table 2:** Work related and clinical characteristics of nurses working at emergency and intensive care unit in Addis Ababa public hospitals, 2017 (*n* = 415)

Variables	Categories	Frequency	Percentage
Current duty shift	Day shift	330	79.5
	Night shift	85	20.4
Presence of work over load	Yes	86	20.7
	No	329	79.3
Job satisfaction	Poor	190	45.8
	Fair	152	36.6
	Good	73	17.6
Plan to leave job	Yes	104	25
	No	228	55.0
	I don’t know	83	20.0
Habit of physical exercise	Yes	89	21.4
	No	326	78.6
Habit of cigarette smoking	Yes	87	20.9
	No	328	79.1
Habit khat chewing	Yes	97	23.4
	No	318	76.6
Habit of alcohol drinking	Yes	86	20.0
	No	329	80.0
Presence of hypertension	Yes	3	0.7
	No	412	99.3

### Level of anxiety

The result of this study shows that 19.8 % nurses working at emergency and intensive care units of Addis Ababa public hospitals had higher level of anxiety symptoms [95 % CI (16.1 %- 23.6 %)].

### Risk factors for anxiety

Binary logistic regression was computed to identify factors associated with higher level of anxiety symptoms. In the bi-variable analysis, marital status, cigarette smoking, Khat chewing, alcohol use, night duty shift and presence of work overload were found to be eligible candidates for multi variable analysis (*P*-value < 0.25) to avoid cofounding variables. In the final model (multi-variable analysis), cigarette smoking, night duty shift and presence of work overload were factors with statistically significant association with higher level of anxiety symptoms among nurses working at emergency and ICU at Addis Ababa public hospitals (Table 3).

**Table 3:** Logistic regression of factors associated with higher level of anxiety symptoms among nurses working at emergency and intensive care units of public hospitals, Addis Ababa, Ethiopia, 2017 (*n* = 415)

Variables	Categories	Level of anxiety symptoms		AOR with 95% CI	*P*- value
		Higher	Lower		
Marital status	Married	57	159	1.00	
	Single	23	170	2.13 (0.31, 5.77)	0.089
	Separated/Divorced	2	4	1.01 (0.98, 8.21)	0.12
Duty shift	Night	9	76	2.41(1.19-6.87)	0.020*
	Day	73	257	1.00	
Khat chewing	Yes	13	84	0.55(.29-1.06)	0.075
	No	69	249	1.00	
Cigarette smoking	Yes	73	78	2.48(1.18-5.18	0.016*
	No	9	255	1.00	1.00
Work over load	Yes	74	78	1.35(1.16,3.76)	0.008**
	No	8	255	1.00	1.00
Alcohol drinking	Yes	51	35	1.32(0.87,1.67)	0.123
	No	31	298	1.00	

*AOR* Adjusted Odds Ratio

Note: *=*P*-value <0.05, **=*P*-value <0.01

## Discussion

The largest workforce in any health care institution is encompassed by nurses as they are dedicated with the care of individuals, families, and communities so they may attain, maintain, or recover optimal health and quality of life [3, 4]. Nurses working at emergency and ICU frequently feel anxiety and dissatisfaction as a result of being a witness for life threatening health conditions and death of clients after many close relation with them. Despite of such public health concerns and negative clinical outcomes of anxiety symptoms, it is not well addressed among health professionals, particular nurses working at emergency health care units. This is the first study conducted in Ethiopia to assess the level of anxiety and its associated factors among nurses working at emergency and ICU at public hospitals.

The findings of this study revealed that 19.8 % of nurses at emergency and ICU showed higher level of anxiety symptoms in Ethiopia [95 % CI (16.1 %- 23.6 %)]. This finding is in line with other similar studies of Greece 21.3 % [18], China11.45 % [18] and Germany 20.0 % [18].

However, a study conducted at Hyderabad hospital reported a higher level (40 %) of anxiety symptoms [19]. This difference could be attributed to the difference of tools used to measure level of anxiety symptoms, differences in sampling techniques, as well as the socio-cultural differences of study participants [20].

On the contrary, the level of anxiety symptoms in the current study was lower than a study in China which revealed that 43 % oncology nurses working in the city general hospitals had high level of anxiety symptoms [21, 22]. The possible explanation for this discrepancy might be due to the difference of measurement scales/tools. The study in China used the Zung Self-Rating Anxiety Scale (SAS) which has a potential to include normal anxiety traits which might over-estimate the level of anxiety symptoms to be higher unlike the tool used in the current study (Hamilton Anxiety Rating Scale).

The second objective of this study was to identify factors associated with higher level of anxiety symptoms among nurses working at emergency and ICU setting of public hospitals. In the multivariable analysis, cigarette smoking, night duty shift and presence of work over-load were found to have statistically significant association with higher levels of anxiety symptoms among nurses working at emergency and ICU medical centers. This finding is supported by other previously conducted studies [22].

The odds of having a higher level of anxiety symptoms among nurses who smoke cigarette were increased by 2.48 times as compared to non-smokers. This might be due to the tension, frustration and irritability effects of Nicotine dependency. Moreover, fear of stigma and discrimination towards people who smokes cigarette, particularly health care professionals may exacerbate anxiety symptoms.

Similarly, the odds of having higher level of anxiety symptoms among nurses with work over-load were found to be increased 1.35 times as compared to their counterparts. This might be due to the physical and mental health effect of prolonged work-related stress. It has also been linked with psychological disturbances, altered professional performance, mediated through emotional exhaustion, vicarious trauma, fatigue and professional burnout work over-load [8–10, 23].

Finally, respondents who were working at night duty shift are 2.41 times more likely to have higher level of anxiety symptoms as compared to nurses who were working at day time shift. This might be explained daytime sleepiness, fear of family separation and problems of psychological adaptation to work at night shift. Other previous researches have also revealed that rotating shifts and sleep quality in nurses has significant negative correlation [12, 13]. This study might have limitations due to the cross section nature of the study design that might not show the cause and effect relationships between variables. In addition, the measurement tool used to screen anxiety symptoms is not validated in Ethiopian context. Therefore, further research works are highly recommended.

## Conclusions

The level of anxiety symptoms among working at emergency and intensive care unit were found to be higher than the general population and nursing working in other medical settings. Cigarette smoking, work overload and night duty shift were found to have statistically significant association with higher level of anxiety symptoms among nurses working at emergency medical settings. This demonstrates a need for the implementation of counseling services and adaptation of effective coping strategies for nurses working at emergency and ICU settings.

## Supplementary Information

**Additional file 1. **Screening tool of anxiety symptoms. Hamilton Anxiety Rating Scale (HAM-A)

## Data Availability

All data included in the manuscript can be accessed from the corresponding author through “zelalembe45@gmail.com”.

## Abbreviations

AA: Addis Ababa
AOR: Adjusted Odds Ratio
HAM-A: Hamilton Anxiety Rating Scale
AS: Anxiety symptoms
ICN: Intensive Care Nurses
ICU: Intensive Care Unit
IRB: Institution Review Board
PI: Principal Investigator
RNs: Register Nurses
SPSS: Statistical Product and Service Solutions
