# Supplementary material for: Level of anxiety symptoms and its associated factors among nurses working in emergency and intensive care unit at public hospitals in Addis Ababa, Ethiopia
Source: BMC Nurs. 2021 Sep 26;20:180. doi: 10.1186/s12912-021-00701-4 (PMC8466700; doi:10.1186/s12912-021-00701-4)
Supplement: Supplementary file 1 — Additional file 1. Screening tool of anxiety symptoms. Hamilton Anxiety Rating Scale (HAM-A) [file 12912_2021_701_MOESM1_ESM.pdf]

Hamilton Anxiety Rating Scale (HAM-A)

Reference: **Hamilton M. The assessment of anxiety states by rating. Br J Med Psychol 1959; 32:50–55.**

Rating Clinician-rated

Administration time 10–15 minutes

Main purpose To assess the severity of symptoms of anxiety

Population Adults, adolescents and children

Commentary

The HAM-A was one of the first rating scales developed to measure the severity of anxiety symptoms, and is still widely used today in both clinical and research settings. The scale consists of 14 items, each defined by a series of symptoms, and measures both psychic anxiety (mental agitation and psychological distress) and somatic anxiety (physical complaints related to anxiety). Although the HAM-A remains widely used as an outcome measure in clinical trials, it has been criticized for its sometimes poor ability to discriminate between anxiolytic and antidepressant effects, and somatic anxiety versus somatic side effects. The HAM-A does not provide any standardized probe questions. Despite this, the reported levels of inter-rater reliability for the scale appear to be acceptable.

Scoring

Each item is scored on a scale of 0 (not present) to 4 (severe), with a total score range of 0–56, where <17 indicates mild severity, 18–24 mild to moderate severity and 25–30 moderate to severe.

Versions

The scale has been translated into: Cantonese for China, French and Spanish. An IVR version of the scale is available from Healthcare Technology Systems.

Additional references

Maier W, Buller R, Philipp M, Heuser I. The Hamilton Anxiety Scale: reliability, validity and sensitivity to change in anxiety and depressive disorders. J Affect Disord 1988;14(1):61–8.

Borkovec T and Costello E. Efficacy of applied relaxation and cognitive behavioral therapy in the treatment of generalized anxiety disorder. J Clin Consult Psychol 1993; 61(4):611–19

Address for correspondence

The HAM-A is in the public domain.

Hamilton Anxiety Rating Scale (HAM-A)

Below is a list of phrases that describe certain feeling that people have. Rate the patients by finding the answer which best describes the extent to which he/she has these conditions. Select one of the five responses for each of the fourteen questions.

0 = Not present, 1 = Mild, 2 = Moderate, 3 = Severe, 4 = Very severe.

1 Anxious mood [0] [1] [2] [3] [4]

Worries, anticipation of the worst, fearful anticipation, irritability.

2 Tension [0] [1] [2] [3] [4]

Feelings of tension, fatigability, startle response, moved to tears easily, trembling, feelings of restlessness, inability to relax.

3 Fears [0] [1] [2] [3] [4]

Of dark, of strangers, of being left alone, of animals, of traffic, of crowds.

4 Insomnia [0] [1] [2] [3] [4]

Difficulty in falling asleep, broken sleep, unsatisfying sleep and fatigue on waking, dreams, nightmares, night terrors.

5 Intellectual [0] [1] [2] [3] [4]

Difficulty in concentration, poor memory.

6 Depressed mood [0] [1] [2] [3] [4]

Loss of interest, lack of pleasure in hobbies, depression, early waking, diurnal swing.

7 Somatic (muscular) [0] [1] [2] [3] [4]

Pains and aches, twitching, stiffness, myoclonic jerks, grinding of teeth, unsteady voice, increased muscular tone.

8 Somatic (sensory) [0] [1] [2] [3] [4]

Tinnitus, blurring of vision, hot and cold flushes, feelings of weakness, pricking sensation.

9 Cardiovascular symptoms [0] [1] [2] [3] [4]

Tachycardia, palpitations, pain in chest, throbbing of vessels, fainting feelings, missing beat.

10 Respiratory symptoms [0] [1] [2] [3] [4]

Pressure or constriction in chest, choking feelings, sighing, dyspnea.

11 Gastrointestinal symptoms [0] [1] [2] [3] [4]

Difficulty in swallowing, wind abdominal pain, burning sensations, abdominal fullness, nausea, vomiting, borborygmi, looseness of bowels, loss of weight, constipation.

12 Genitourinary symptoms [0] [1] [2] [3] [4]

Frequency of micturition, urgency of micturition, amenorrhea, menorrhagia, development of frigidity, premature ejaculation, loss of libido, impotence.

13 Autonomic symptoms [0] [1] [2] [3] [4]

Dry mouth, flushing, pallor, tendency to sweat, giddiness, tension headache, raising of hair.

14 Behavior at interview [0] [1] [2] [3] [4]

Fidgeting, restlessness or pacing, tremor of hands, furrowed brow, strained face, sighing or rapid respiration, facial pallor, swallowing, etc.
